# A novel meta learning based stacked approach for diagnosis of thyroid syndrome

**DOI:** 10.1371/journal.pone.0312313

**Published:** 2024-11-01

**Authors:** Muhammad Asad Abbas, Kashif Munir, Ali Raza, Madiha Amjad, Nagwan Abdel Samee, Mona M. Jamjoom, Zahid Ullah

**Affiliations:** 1 Institute of Information Technology, Khwaja Fareed University of Engineering and Information Technology, Rahim Yar Khan, Pakistan; 2 Department of Software Engineering, University Of Lahore, Lahore, Pakistan; 3 Department of Information Technology, College of Computer and Information Sciences, Princess Nourah bint Abdulrahman University, Riyadh, Saudi Arabia; 4 Department of Computer Sciences, College of Computer and Information Sciences, Princess Nourah bint Abdulrahman University, Riyadh, Saudi Arabia; 5 Information Systems Department, College of Computer and Information Sciences, Imam Mohammad Ibn Saud Islamic University (IMSIU), Riyadh, Saudi Arabia; Sreenidhi Institute of Science and Technology, INDIA

## Abstract

Thyroid syndrome, a complex endocrine disorder, involves the dysregulation of the thyroid gland, impacting vital physiological functions. Common causes include autoimmune disorders, iodine deficiency, and genetic predispositions. The effects of thyroid syndrome extend beyond the thyroid itself, affecting metabolism, energy levels, and overall well-being. Thyroid syndrome is associated with severe cases of thyroid dysfunction, highlighting the potentially life-threatening consequences of untreated or inadequately managed thyroid disorders. This research aims to propose an advanced meta-learning approach for the timely detection of Thyroid syndrome. We used a standard thyroid-balanced dataset containing 7,000 patient records to apply advanced machine-learning methods. We proposed a novel meta-learning model based on a unique stack of K-Neighbors (KN) and Random Forest (RF) models. Then, a meta-learning Logistic Regression (LR) model is built based on the collective experience of stacked models. For the first time, the novel proposed KRL (KN-RF-LR) method is employed for the effective diagnosis of Thyroid syndrome. Extensive research experiments illustrated that the novel proposed KRL outperformed state-of-the-art approaches, achieving an impressive performance accuracy of 98%. We vindicated the performance scores through k-fold cross-validation and enhanced performance using hyperparameter tuning. Our research revolutionized the timely detection of thyroid syndrome, contributing to the enhancement of human life by reducing thyroid mortality rates.

## Introduction

Diagnosing thyroid syndrome is challenging, involving multiple processes [1]. The typical procedure comprises a thorough physical examination and various blood tests [2]. Therefore, there is a need to develop a model capable of diagnosing thyroid disease in its early stages. Thyroid diseases encompass a spectrum of conditions that affect the thyroid gland [3], a small yet vital organ located in the neck. The thyroid gland is responsible for producing hormones that regulate essential bodily functions such as metabolism, heart rate, and temperature regulation in diseases.

Thyroid Disease is a significant field within endocrinology and holds a crucial position in medical science [4]. There are two primary types of thyroid diseases: hypothyroidism and hyperthyroidism [5]. In thyroid disease, the levels of chemicals such as TSH can either increase or decrease. When TSH levels decrease, it is referred to as hyperthyroidism, and when they are elevated, it is termed hypothyroidism. In hyperthyroidism, patients commonly experience persistent fatigue, heightened body temperature, increased heart rate, and mood swings. Weight loss is also a common symptom. On the other hand, in hypothyroidism [6], patients often complain of fatigue, slowed cognitive function, sensitivity to colds, increased sleep duration, weight gain, and feelings of sadness.

Thyroid disease occurs in individuals who are deficient in iodine; hence, many experts recommend using iodized salt to prevent goiter [7]. Those who consume a significant amount of vegetables, such as cabbage or spinach, are at a higher risk of developing goiter, and pregnant women are also more susceptible. When there is an overproduction of thyroid hormone by the thyroid gland, it results in hyperthyroidism, leading to a rapid depletion of the body’s energy resources. This condition not only results in fatigue but can also accelerate heart rate, impede weight loss, and contribute to irritability. Thyroid disorders are prevalent worldwide, particularly among women, with more than 200 million people currently affected [8]. The prevalence of thyroid disorders varies across different regions and populations.

Machine learning in medicine plays an important role in thyroid diagnosis [9], offering numerous classification models that enable the training of accurate models using appropriate data from thyroid patients, ultimately leading to precise predictions. This research introduces a novel meta-learning-based stacked model for the diagnosis of thyroid syndrome. The meta-learner effectively trains on a stack of two machine-learning models, significantly enhancing performance accuracy.

Our innovative research study brings forth the following significant contributions:

- A novel meta-learning model, KRL, is proposed, which is based on a unique stack of K-Neighbors (KN) and Random Forest (RF) models. Subsequently, a meta-learning Logistic Regression (LR) model is constructed using the collective experience of the stacked models.
- We applied four advanced machine learning models in comparisons. The performance of the applied methods is enhanced using hyperparameter optimization tuning and vindicated using k-fold cross-validation.

The rest of the study is categorized as Section “Literature Review”, which contains the analysis of limitations in the literature research. Section “Proposed Methodology” illustrates the proposed study methodology of this research. The results of used machine learning approaches are comparatively evaluated in Section “Results and discussions”. The findings of this innovative research study are summarized in Section “Conclusion and future direction”.

## Literature review

This literature review section for thyroid syndrome detection using machine learning delves into existing research, providing a comprehensive overview of methodologies and advancements in the field. Synthesizing findings from various studies, this section highlights the evolving landscape of machine learning applications for thyroid disorder diagnosis, shedding light on both challenges and breakthroughs, as compared in Table 1.

**Table 1:** The research limitations and summary analysis.

Ref.	Year	Dataset	Technique	Performance Score
[10]	2021	Data of AOU Federico II hospital of Nepalese	Extra Tree Classifier	84%
[11]	2022	UCI repository	Random forest algorithm	94%
[12]	2016	Data of K.N Toosi University of Technology, imam Khomeini hospital	Support vector machine	93%
[13]	2019	General Electrics, USA	Artificial Neural Network	90%
[14]	2023	Data which contained 816 proteins sequence	Random forest algorithm	94%
[15]	2022	dataset containing a total of 7000 lines	k-Nearest Neighbors	95%
[16]	2020	Dataset is taken from Kaggle	Decision tree	95%
[17]	2020	7200 instances and 27 attributes	Support Vector Machine	92%

The study [11] sought to forecast trends in LT4 treatment for patients with hypothyroidism. To achieve this goal, a dedicated database was established, encompassing medical data from individuals receiving treatment at the ‘AOU Federico II hospital in Naples. The temporal availability of each patient’s medical history facilitated a comprehensive analysis, enabling the understanding and prediction of whether treatment adjustments were warranted based on fluctuations in hormonal and other requirements. Various machine learning algorithms were employed to conduct the research, with a specific focus on comparing the outcomes of 10 different models. The performance of these diverse algorithms yielded promising results, with the Extra Tree Classifier standing out by achieving an accuracy rate of 84%.

The triage time significantly impacted the timely treatment of patients [10]. Therefore, the automatic and accurate detection of thyroid nodules in ultrasound images is crucial for reducing the workload and minimizing errors among radiologists. Medical images have transformed into a highly valuable and regularly employed type of data within the field of machine learning applications. This research systematically examined different machine learning methodologies, encompassing decision trees, random forests, K-nearest neighbors (KNN), and adverse energy resources. The investigation also considered specific dataset attributes, such as the lack of constructive algorithms, aiming to improve the accuracy of disease prediction. Furthermore, the dataset underwent thorough analysis to estimate its distribution precisely. To facilitate a more meaningful comparison, sampled datasets and unsampled datasets were categorically differentiated. Following dataset manipulation, the random forest algorithm emerged as the most accurate, achieving a remarkable accuracy rate of 94.8%, while an accuracy rate of 91% was attained overall.

The research [13] plan aimed to engage specialists to investigate the progression of diseases involving the thyroid gland. The resources essential for undertaking this investigation were obtained from the Intelligent Systems Laboratory at K.N. Tusi University of Science and Technology and Imam Khomeini Hospital. Initially, a matching sequence (SMS) was formulated to predict Thyroid Disease Development (TDD) using available data. In cases where SMS failed, the design yielded experimental results of 65% and 93% accuracy through manual optimization and optimized separation methods, respectively.

This study [12] employed machine learning-based methods, specifically utilizing support vectors, artificial neural networks, and random forest classifiers, for the classification of thyroid tissues. The classifier was trained using a novel approach proposed by the research group. In this method, autoregressive models were applied to the signal version of 2D thyroid ultrasound (US) images, resulting in the calculation of 30 spectral power-based features. These features were then utilized to distinguish between thyroid and non-thyroid tissues. Notably, this approach diverged from conventional methods in the literature by incorporating image-based features in the generation of thyroid tissue classifications. The combination of these three methods yielded an impressive accuracy rate of approximately 90%.

A computer-aided diagnosis system employing machine learning within the framework of multiple instance learning (MIL) was designed [18], with a specific emphasis on the classification of benign and malignant instances. The dataset used for this research study was sourced from the National University of Colombia and was partitioned into an 87% training set and a 13% validation set. The study compared the performance of Artificial Neural Network (ANN) and Support Vector Machine (SVM) classification algorithms, evaluating them based on sensitivity, accuracy, and specificity scores. The obtained results revealed an accuracy of 75% for ANN and 96% for SVM.

The proposed method [14], employing the Random Forest algorithm enhanced by AdaBoost, achieved an impressive accuracy of 94.37% and an F1-Score of 0.94 in predicting High Blood Pressure (HBP) during 5-fold cross-validation. This underscores the robustness of machine learning in handling dissimilar sequences and highlights its pivotal role in advancing protein function prediction, particularly in understanding hormone regulation mechanisms.

The study [15] on the rapid and accurate diagnosis of thyroid disease, a prevalent global concern, has been pivotal for effective healthcare management. Leveraging machine learning techniques to diagnose thyroid ailments offered a promising avenue, considering the dataset’s 7000 entries encompassing patient information and test results. Strategies such as correlation-based feature extraction, undersampling, and oversampling were employed to address imbalanced distributions and overfitting. The identification and removal of non-influential parameters further refined the dataset. Results indicated that feature extraction via correlation had proved to be the most effective. Assessment using support vector machines, logistic regression, k-nearest neighbors, and decision trees, with a focus on the random forest algorithm, demonstrated that artificial neural networks, k-nearest neighbors, and support vector machines emerged as the most effective classifiers in addressing this multi-classification challenge. These findings highlight their potential for precise diagnosis of thyroid diseases.

The application of machine learning in medical diagnostics [17], particularly in ailments such as thyroid disease, has been pivotal for early detection and precise treatment. Traditional diagnostic methods involved meticulous examination and blood tests, aiming for accurate early-stage identification. Machine learning techniques have significantly contributed to correct decision-making, efficient disease diagnosis, and substantial savings in patient time and costs within the medical field. The research objective was to anticipate thyroid disorders using classification predictive modeling. Decision Tree ID3 and Naive Bayes algorithms were applied to analyze the Thyroid Patient dataset. The Decision Tree algorithm assessed the likelihood of thyroid presence in patients, and the subsequent application of the Naive Bayes algorithm determined the stage of thyroid disease in cases where it was identified.

The research [17] delved into various feature selection and classification methods for diagnosing thyroid diseases, which are pivotal in machine-learning classification. Hyperthyroidism and hypothyroidism, prevalent thyroid disorders that impact metabolic rates, presented a notable classification challenge. Feature selection, a critical step in pattern recognition, encompasses techniques such as Recursive Feature Elimination, Univariate Selection, and Tree-Based Feature Selection. By employing classification techniques like Naive Bayes, Support Vector Machines, and Random Forest, the study determined that Support Vector Machines exhibited the highest accuracy. As a result of these discoveries, the utilization of Support Vector Machines was endorsed for the categorization of symptoms associated with thyroid disease into four specific classes: Hyperthyroid, Hypothyroid, Sick Euthyroid, and Euthyroid (negative).

This research [19] aimed to properly classify brain tumours by artificial intelligence, which was critical for early detection and treatment. The study trained deep learning models on classified gene expression data of brain tumors to distinguish tumor classes. A new optimization method called PSCS (Particle Swarm Optimization combined with Cuckoo Search) was proposed to boost the accuracy of deep learning-based brain tumor classification. Similarly, in another research [20], redundancy and irrelevant features in high-dimensional biological data made it challenging to identify significant genes. The authors used a dataset related to breast cancer that incorporates gene expression profiles and clinical outcomes to test the performance of the SCACSA algorithm in performing gene selection. Another study [21] aimed at the decentralized data storage and unstructured (a mix of structured and unstructured) logs and developed the forensic framework for wide column store NoSQL databases. The authors examined three datasets: synthetic, real-world, and experimental datasets from NoSQL databases, like user activities, transaction logs and system metadata.

## Proposed methodology

This segment thoroughly examines our innovative approach to identifying thyroid syndrome in patients. We employed various cutting-edge machine learning models and assessed their performance metrics. The dataset used, and all its related preprocessing steps are described. This section shows the proposed methodology’s stepwise architectures.

**Fig 1:**
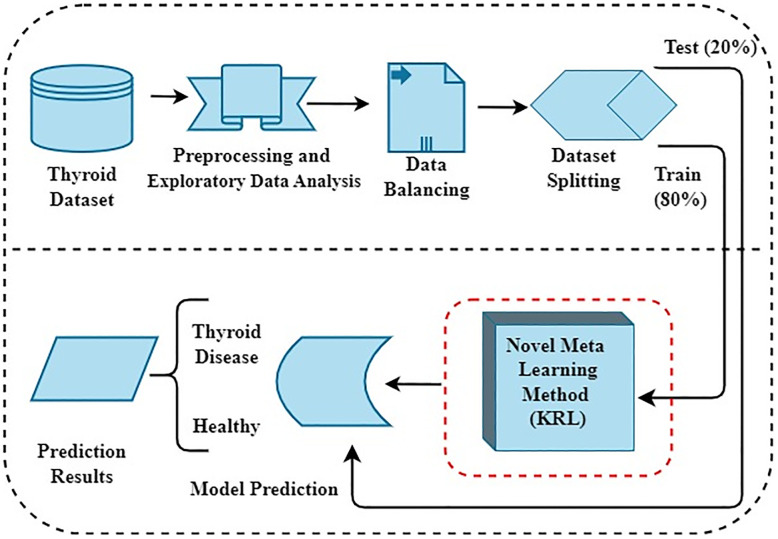
The novel proposed methodology workflow analysis.

Fig 1 presents our innovative research workflow designed to detect thyroid syndrome. We have used standard thyroid patient data to conduct our research experiments. Some preprocessing steps are initially applied to remove noise, and Exploratory Data Analysis (EDA) is performed. Subsequently, we identify dataset imbalance. To address this issue, we employ SMOTE techniques for dataset balancing. The evenly distributed dataset is subsequently split into training and testing subsets using an 80% to 20% ratio. We implement several advanced machine learning methods on the dataset, and hyperparameter tuning is performed to enhance performance scores. Finally, our novel proposed meta-learning model is utilized to recognise thyroid syndrome in patients.

### Thyroid patients data

The standard thyroid dataset, consisting of 3,772 patient records, is obtained from Kaggle [22]. The dataset file typically includes essential patient details such as age, sex, referral source, and more. This information about patients is stored in the dataset and utilized as patient information for subsequent thyroid diagnoses. The dataset highlights factors with a higher likelihood of causing thyroid disease and disregards others, represented by a Boolean value indicating positive or negative outcomes. By considering the patient’s historical medical records and descriptive information, the aim of this research is to enhance the accuracy of results, thereby facilitating more effective diagnoses by medical personnel.

### Data pre-processing and EDA

The noise in the dataset can lead to errors when running algorithms or models. Therefore, the analysis of data in high-dimensional data space requires more time for processing, especially in the case of high-end products. Initially, we preprocessed the data to remove noise from it. We filled null values with the mean of the data. The Boolean columns are encoded with numeric values. The cleaned dataset is then utilized to conduct the next steps of the proposed research methodology.

**Fig 2:**
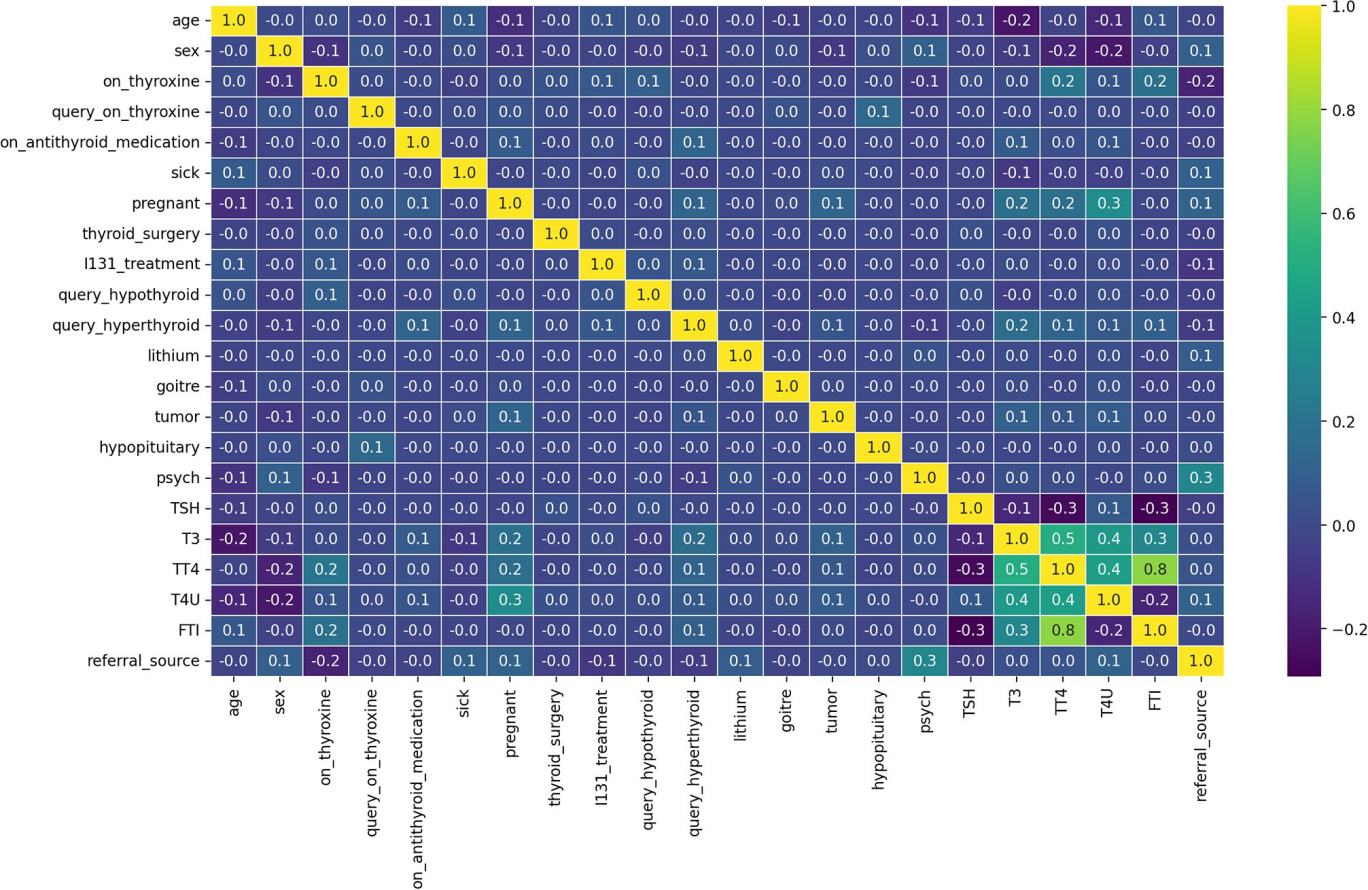
The heatmap-based correlation analysis.

In addition to Exploratory Data Analysis (EDA), we have illustrated the heatmap-based correlation diagrams of features. The analysis is shown in Fig 2. The analysis demonstrates that T3, TT4, T4U, and FTI have high positive correlation values. Only the TSH and sex features have slightly low negative correlation values. The analysis concludes that these features exhibit good correlations among themselves, which will contribute to achieving high-performance scores.

### SMOTE based data over sampling

**Fig 3:**
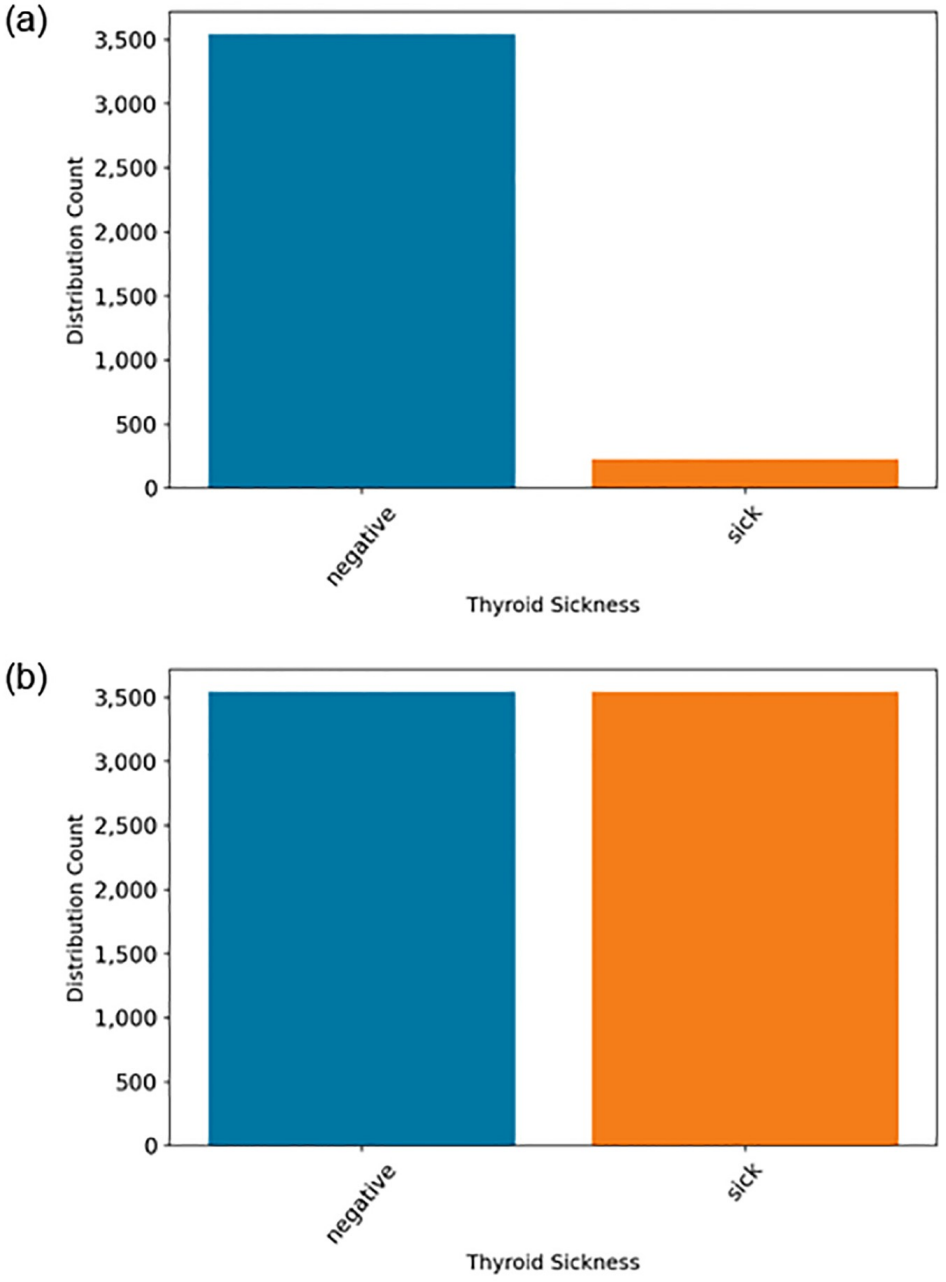
The oversampling dataset analysis.

After preprocessing the dataset, we discovered that the dataset exhibits an imbalance issue. Dataset imbalance occurs when the distribution of classes is not equal, meaning that one class has significantly fewer instances than others. When there is a class imbalance, models tend to be biased towards the majority class, as it is more frequently encountered during training. This bias can result in the model having a poor ability to predict the minority class. To address this issue, we employed the SMOTE technique [23] to oversample the dataset. The results before and after oversampling are illustrated in Fig 3. The balanced 7,000 patient records are utilized for training applied machine learning methods.

### Data splitting

In this research study, an 80:20 split data ratio is employed for data distribution [24]. Eighty percent of the dataset is allocated for machine learning training, while the remaining 20% of data is reserved for evaluating the model’s effectiveness. Dataset splitting is accomplished using the train_test_split() function from the scikit-learn module, addressing overfitting concerns and enhancing the model’s capabilities. Additionally, the utilization of k-fold cross-validation is employed to partition the data for result validation.

### Applied machine learning approaches

Machine learning approaches for thyroid detection have gained prominence in medical research [25, 26], offering automated methods to enhance the accuracy of thyroid disease diagnosis. Leveraging diverse datasets, these approaches employ algorithms to analyze medical imaging, clinical data, and patient records, contributing to the early and precise detection of thyroid abnormalities.

#### Random Forest Classifier

The Random Forest Classifier (RFC) [27, 28] is a classification and regression technique that utilizes multiple decision trees, employing methods like bootstrapping to create a forest. It represents a fusion of tree predictions, with each tree relying on the selected value of a vector. Upon receiving fresh input information, the algorithm creates a decision tree specific to the data and integrates it into a forest along with supplementary decision trees [29–31]. RFC for classification include its high accuracy and robustness to overfitting due to the ensemble of decision trees. However, drawbacks include its complexity, computational intensity, and less interpretability compared to simpler models like logistic regression. The basic mathematical equation for RFC classification is given by:

$$\begin{array}{c} \hat{y}=\text{mode}({\hat{y}}_{1},{\hat{y}}_{2},\ldots ,{\hat{y}}_{n}) \end{array}$$ (1)

Here, $\hat{y}$ is the predicted data class, and ${\hat{y}}_{1},{\hat{y}}_{2},\ldots ,{\hat{y}}_{n}$ are the predicted classes from each used decision trees. The mode function selects the most frequently occurring class among the predictions.

#### Logistic Regression

Logistic Regression (LR) [32, 33] is a widely utilized method in machine learning for predicting binary values by estimating the probability that inputs belong to a specific category. Through standard training procedures, which adjust weights to minimize the error in predicting outcomes against actual results, the model learns to discern differences between groups. LR for classification includes its simplicity and efficiency in binary classification problems and its ability to provide probabilistic interpretations of class memberships. However, it may struggle with complex relationships in data due to its linear decision boundary. The mathematical expressions derivations of the LR model are expressed as:

$$\begin{array}{c} P\left(Y=1|X\right)=\frac{1}{1+e^{-(\beta_{0}+\beta_{1}X_{1}+\beta_{2}X_{2}+\ldots +\beta_{n}X_{n})}} \end{array}$$ (2)

#### K-Neighbor Classifier

The K-Neighbor Classifier (KNC) [34, 35] is a classification algorithm that predicts the label of new data by identifying the most common class of nearest neighbors at a given location. It utilizes a distance metric to measure similarity and does not construct models. KNC is advantageous due to its simplicity and effectiveness in handling non-linear data, but it can be computationally expensive and sensitive to the choice of k and feature scaling. The simple basic mathematical equation for the KNC method is given below:

$$\begin{array}{c} y\left(x\right)=\text{mode}{\left\{y_{i}\right\}}_{i\in N_{k}\left(x\right)}, \end{array}$$ (3)

#### Multi-Layer Perceptron Classifier

The Multi Layer Perceptron (MLP) Classifier [36, 37] in machine learning refers to a multilayer perceptron classifier, which is a potent neural network model exclusively employed for task classification. Numerous layers of connections exist between nodes or neurons, and the model operates in a feed-forward manner, with information passing through each layer and establishing connections within and between layers. MLP for classification include its ability to model complex, non-linear relationships in data. However, MLPs can be computationally intensive and prone to overfitting, requiring careful tuning of hyperparameters. The MLP Classifier method for classification can be mathematically represented as follows:

$$\begin{array}{c} \text{Input}\;\text{Layer:}\;X=[\begin{array}{c} x_{1}\\ x_{2}\\ \vdots \\ x_{n} \end{array}] \end{array}$$ (4)

$$\begin{array}{c} \text{Hidden}\;\text{Layer}\;\text{Activation:}\;A^{\left[l\right]}=g^{\left[l\right]}\left(W^{\left[l\right]}A^{\left[l-1\right]}+b^{\left[l\right]}\right) \end{array}$$ (5)

$$\begin{array}{c} \text{Output}\;\text{Layer}\;\text{Activation:}\;\hat{Y}=g^{\left[L\right]}\left(W^{\left[L\right]}A^{\left[L-1\right]}+b^{\left[L\right]}\right) \end{array}$$ (6)

### Novel proposed meta-learning approach

**Fig 4:**
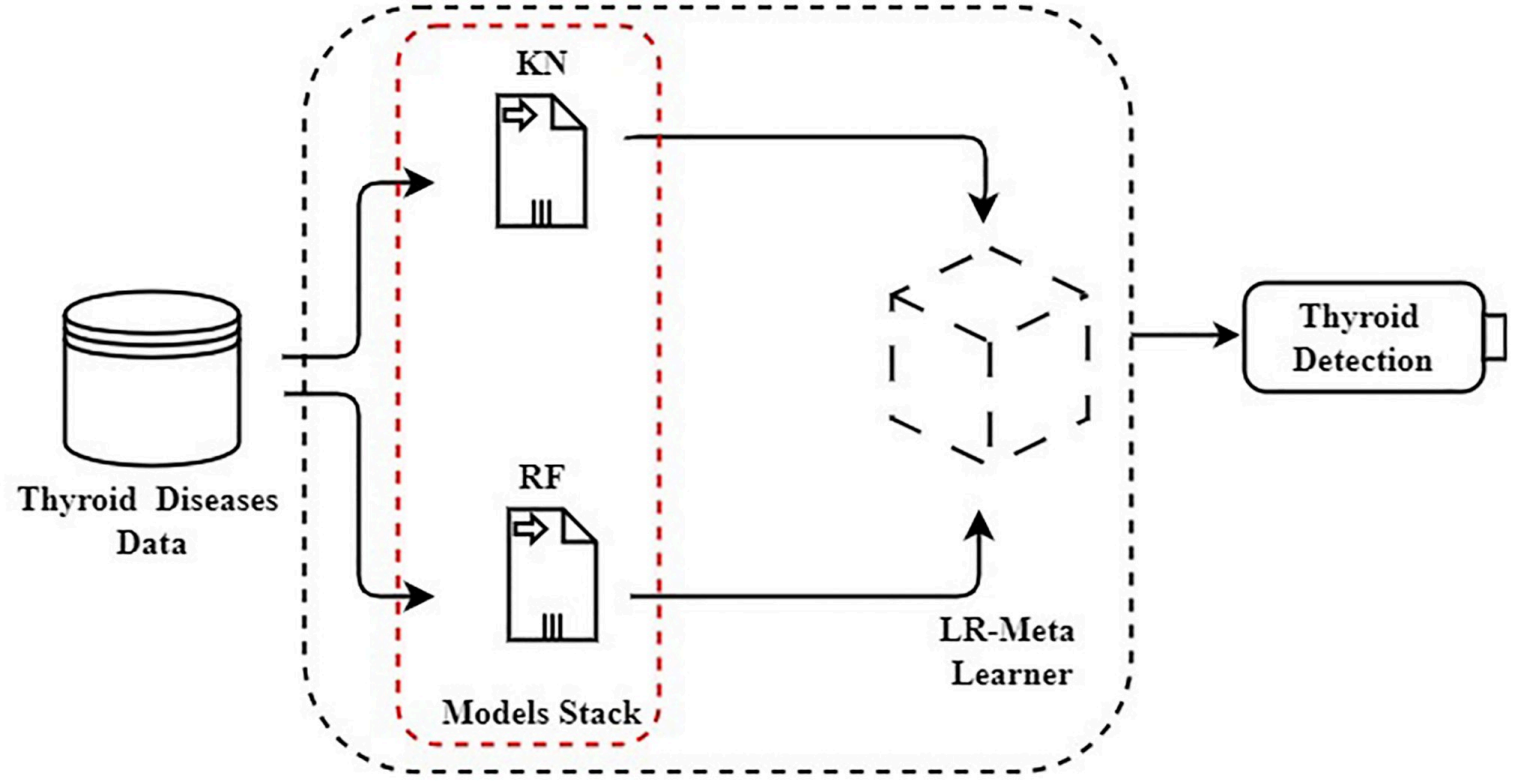
The proposed KRL approach architectural analysis.

A novel meta-learning approach, KRL (KN-RF-LR), is proposed for diagnosing thyroid disease. The preprocessed dataset is inputted into KNN and RF methods for prediction, as illustrated in Fig 4. The prediction outputs from these two separate groups are then combined and fed into a logistic-based unique meta-learner to generate the final class prediction. The LR-based meta-learner capitalizes on the strengths of each method in the set by utilizing their outputs as inputs. Experimental performance results demonstrate that the KRL scheme scores high performance in diagnosing thyroid disease.

The novel proposed meta-learning classifier is represented by:

$$\begin{array}{cc} \text{RF:}\; & {\hat{y}}_{\text{RF}}=\text{RF}\left(\text{input\_data}\right)\\ \text{KNC:}\; & {\hat{y}}_{\text{KNN}}=\text{KNN}\left(\text{input\_data}\right) \end{array}$$

The outputs from the base classifiers are combined and fed into the meta-classifier LR:

$$\begin{array}{c} \text{Combined}\;\text{Input}\;\text{for}\;\text{LR:}\;X_{\text{LR}}=\left[{\hat{y}}_{\text{RF}},{\hat{y}}_{\text{KNC}}\right] \end{array}$$ (7)

The final class prediction of the meta-learning method is given by:

$$\begin{array}{c} \text{Final}\;\text{Prediction:}\;{\hat{y}}_{\text{final}}=\text{LR}\left(X_{\text{LR}}\right) \end{array}$$ (8)

The term “meta” typically signifies something more comprehensive or abstract. For instance, a metaverse is a virtual universe within our world, and metadata is information about other data. In a similar vein, meta-learning refers to the concept of learning about learning. This involves machine learning algorithms that learn from the outputs of other machine learning algorithms. In traditional machine learning, we focus on identifying the algorithms that perform best with our data. These algorithms learn from historical data to build models, which are then used to make predictions for thyroid detection. In contrast, meta-learning algorithms do not directly use historical data. Instead, they learn from the outputs of machine learning models. Therefore, meta-learning algorithms require pre-trained models.

The algorithmic notations of our proposed meta-learning approach are described in Algorithm 1.

**Algorithm 1** Novel KRL Algorithm

1: **procedure** MetaLearning(input_data)

2:  ${\hat{y}}_{\text{RF}}\leftarrow \text{RF}\left(\text{input\_data}\right)$    ▷ Random Forest base classifier

3: ${\hat{y}}_{\text{KNN}}\leftarrow \text{KNN}\left(\text{input\_data}\right)$    ▷ K-Nearest Neighbors base classifier

4: $X_{\text{LR}}\leftarrow \left[{\hat{y}}_{\text{RF}},{\hat{y}}_{\text{KNN}}\right]$    ▷ Combine outputs for LR

5: ${\hat{y}}_{\text{final}}\leftarrow \text{LR}\left(X_{\text{LR}}\right)$    ▷ Logistic Regression meta-classifier

6:  **return** ${\hat{y}}_{\text{final}}$    ▷ Final prediction

7: **end procedure**

### Hyperparameter settings choices

The hyperparameter tuning analysis is presented in Table 2. This examination delves into the hyperparameters of each applied model to achieve optimal performance by carefully selecting the most important features in a machine-learning model for predicting thyroid disease. We employed k-fold cross-validations and recursive propagation of test and training sets to identify the optimal parameters. By identifying the most effective hyperparameters through back-training and validation techniques, the technology becomes more effective, resulting in improved scores for identifying thyroid diseases.

**Table 2:** Optimizing hyperparameters in used machine learning models.

Model	Parameters and description
LR	random_state = 0, test_size = 0.2, random_state = 42, C = 1.0, random_state = Non, warm_start = False
RFC	n_estimators = 2, max_depth = 2, random_state = 0, criterion = ‘gini’, verbose = 0, class_weight = None, multi_class = ‘auto’
KNC	n_neighbors = 3, test_size = 0.2, random_state = 42,algorithm = ‘auto’, weights = ‘uniform’
MLP	random_state = 1, max_iter = 30, test_size = 0.2, random_state = 42,nesterovs_momentum = True, power_t = 0.5

## Results and discussions

This section is dedicated to investigating the outcomes and discourse surrounding machine learning models utilized in the diagnosis of thyroid conditions. The study used different data including thyroid disease in various conditions to evaluate the effectiveness of the model. The findings and analysis presented in this research illustrate the efficacy of employing machine learning models for the classification of thyroid diseases.

### Experimental setup

The utilization of machine learning models and deep learning models involves the creation of advanced programming constructs in Python, specifically in version 3.6 of the language. The pandas module is utilized to import and analyze the thyroid dataset file. The investigations were carried out utilizing Google Colab [38] on hardware equipped with a GPU backend, 13 GB RAM, and 90 GB of disk space. Performance evaluation of the employed machine learning techniques was conducted using metrics such as accuracy, precision, recall, and F1 scores.

### Results of applied methods

The performance results of applied advanced machine learning methods for the diagnosis of thyroid are analyzed in this section. The assessment of the performance metrics for each employed method is conducted utilizing undisclosed test data.

The performance results using unseen test data for the applied methods are described in Table 3. We have evaluated the performance using accuracy, F1, precision, and recall scores for the applied methods. The analysis demonstrates that the KNC approach shows a lower performance accuracy of 0.91 in comparison with others. The MLP approach achieved a high accuracy of 0.95 in this analysis. However, there is still a high loss error observed in the model evaluations. To enhance performance scores for thyroid diagnosis, there is a need for a more advanced learning approach.

**Table 3:** The performance comparisons of used methods with unseen testing data.

Technique	Accuracy	Precision	Recall	F1
LR	0.92	0.92	0.92	0.92
RFC	0.94	0.94	0.94	0.94
KNC	0.91	0.91	0.90	0.91
MLP	0.95	0.95	0.95	0.95

**Fig 5:**
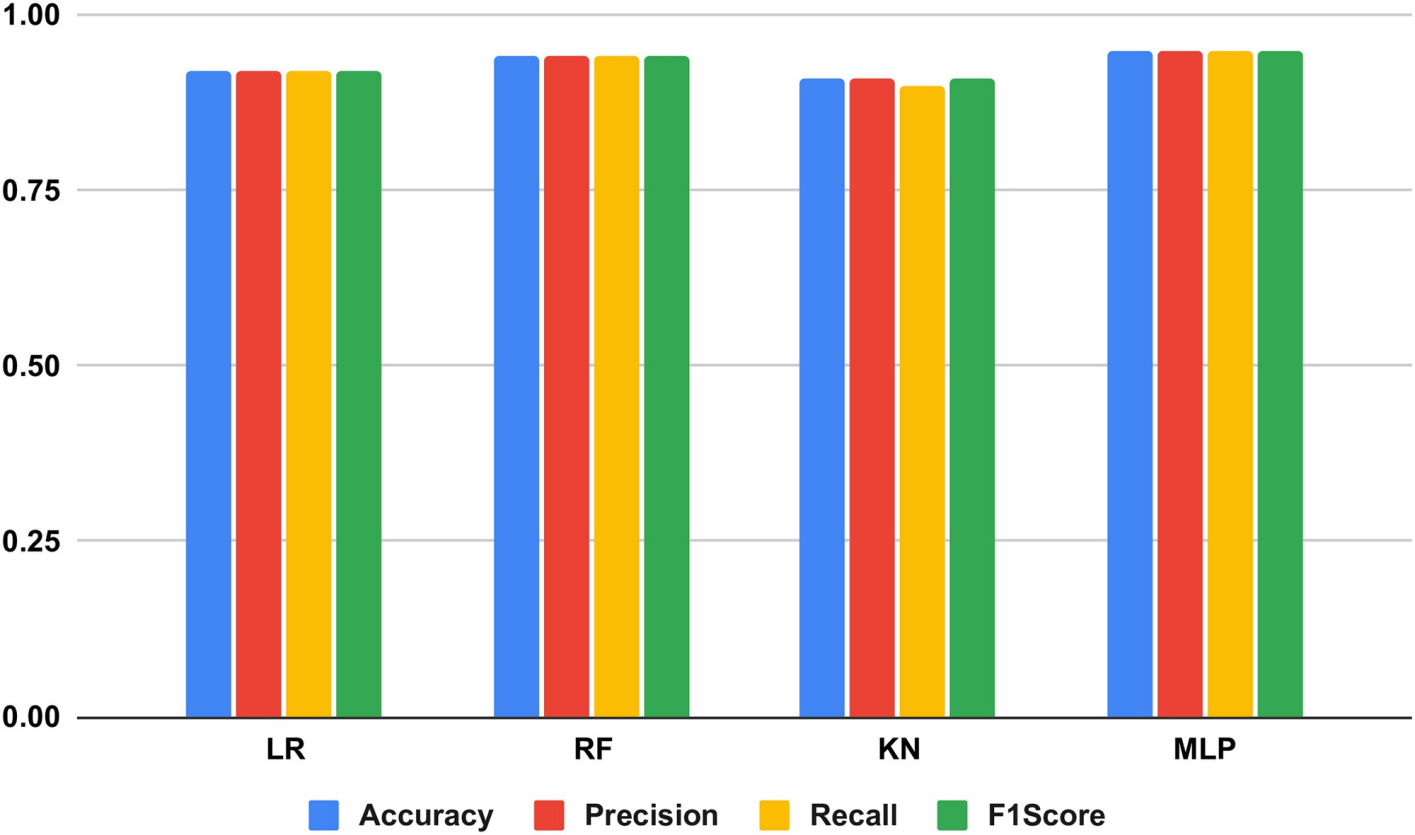
Analyzing the performance of employed techniques through a histogram chart.

The performance analysis of the applied methods is illustrated in the histogram chart shown in Fig 5. This analysis compares the scores of all performance metrics and visualizes them in different comparison colours. The examination reveals that the utilized KNC method exhibited inferior results across all metrics in comparison to alternative approaches. The overall analysis shows that MLP and RFC achieved good scores in comparisons.

For a more detailed performance analysis, we have evaluated the performance results for each class in the dataset. The class-wise accuracy results are reported in Table 4. The analysis demonstrates that a low precision score of 0.87 is achieved for the ‘sick’ class using the KNC approach. This is the main reason KNC achieved lower scores in comparison. All remaining models achieved good class-wise performance scores, which are above 0.90 in this analysis.

**Table 4:** Analyzing the performance of methods applied across different classes.

Models	Accuracy	Target Class	Precision	Recall	F1Score
LR	0.92	Negative	0.92	0.93	0.92
		Sick	0.93	0.92	0.92
		Average	0.92	0.92	0.92
RFC	0.94	Negative	0.93	0.96	0.94
		Sick	0.96	0.92	0.94
		Average	0.94	0.94	0.94
KNC	0.91	Negative	0.95	0.85	0.90
		Sick	0.87	0.96	0.91
		Average	0.91	0.90	0.91
MLP	0.95	Negative	0.98	0.91	0.95
		Sick	0.92	0.98	0.95
		Average	0.95	0.95	0.95

**Fig 6:**
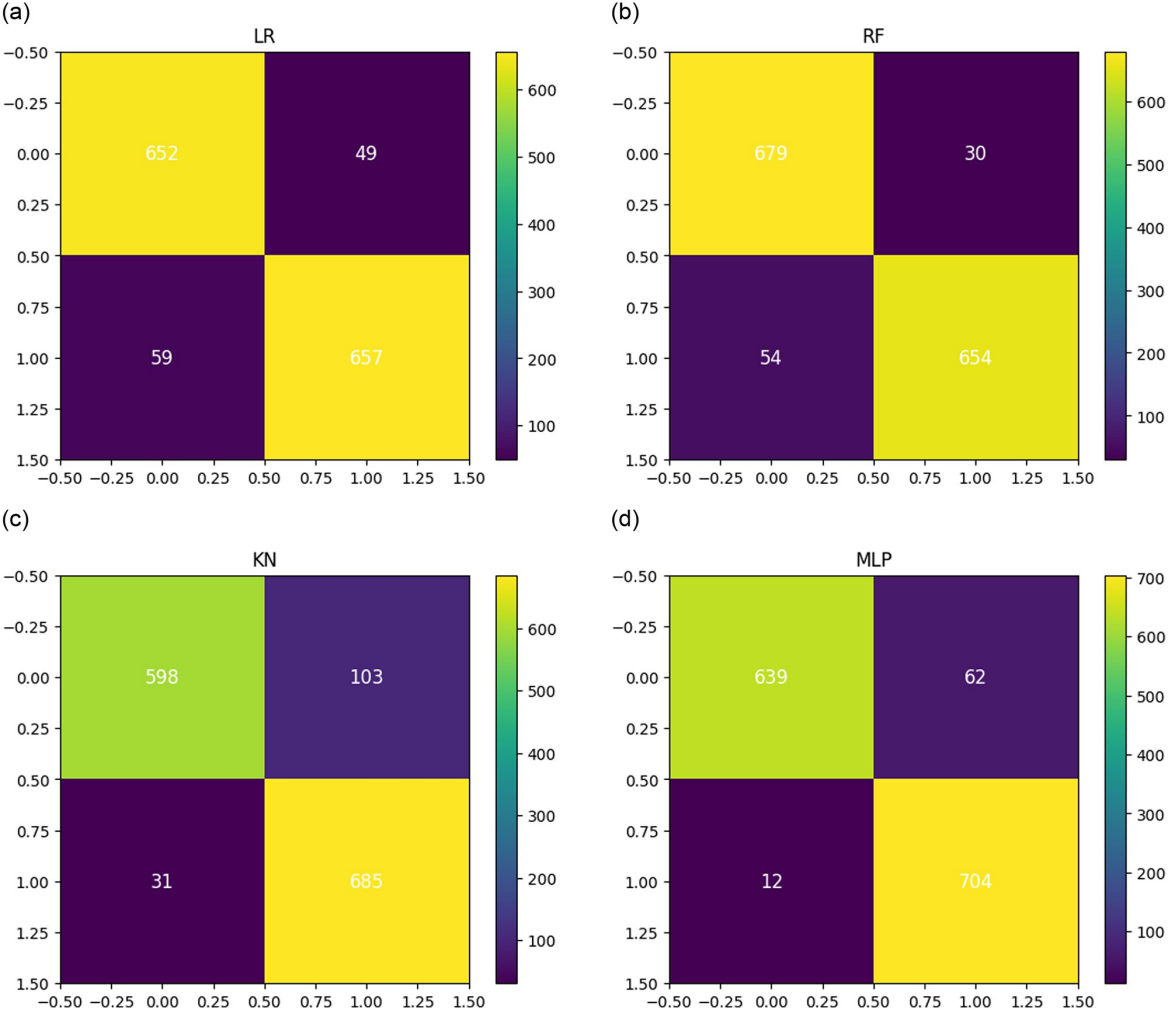
The confusion matrix analysis of applied machine learning methods.

The depiction of the confusion matrix analysis for performance validations using various machine learning techniques is presented in Fig 6. The analysis described that the KNC predicts 134 incorrect outputs, RF predicts 84 erroneous outputs, and the LR approach scores 108 wrong outputs. This indicates that the applied methods have high loss errors for the testing data. The MLP model achieved 74 incorrect predictions as output. This analysis concludes that there are still low-performance scores using the applied methods.

### Results of novel proposed method

Finally, our novel proposed KRL results are analyzed in this section. We have evaluated the performance accuracy and class-wise results of the proposed approach, as illustrated in Table 5. The proposed model achieves good accuracy with a recall, precision, and an f1 score of 0.98. This shows that the KRL method is very useful in identifying thyroid problems of different severity, including Negative, Sick, and Average. Overall, the meta-analysis demonstrates the potential of diagnosing thyroid disease with high results and performance scores.

**Table 5:** The performance results analysis of novel proposed KRL approach for unseen testing data.

Model	Accuracy	Target Class	Precision	Recall	F1Score	AUC	MCC
KRL	0.98	Negative	0.98	0.98	0.98	0.9995	0.9693
		Sick	0.98	0.98	0.98		
		Average	0.98	0.98	0.98		

**Fig 7:**
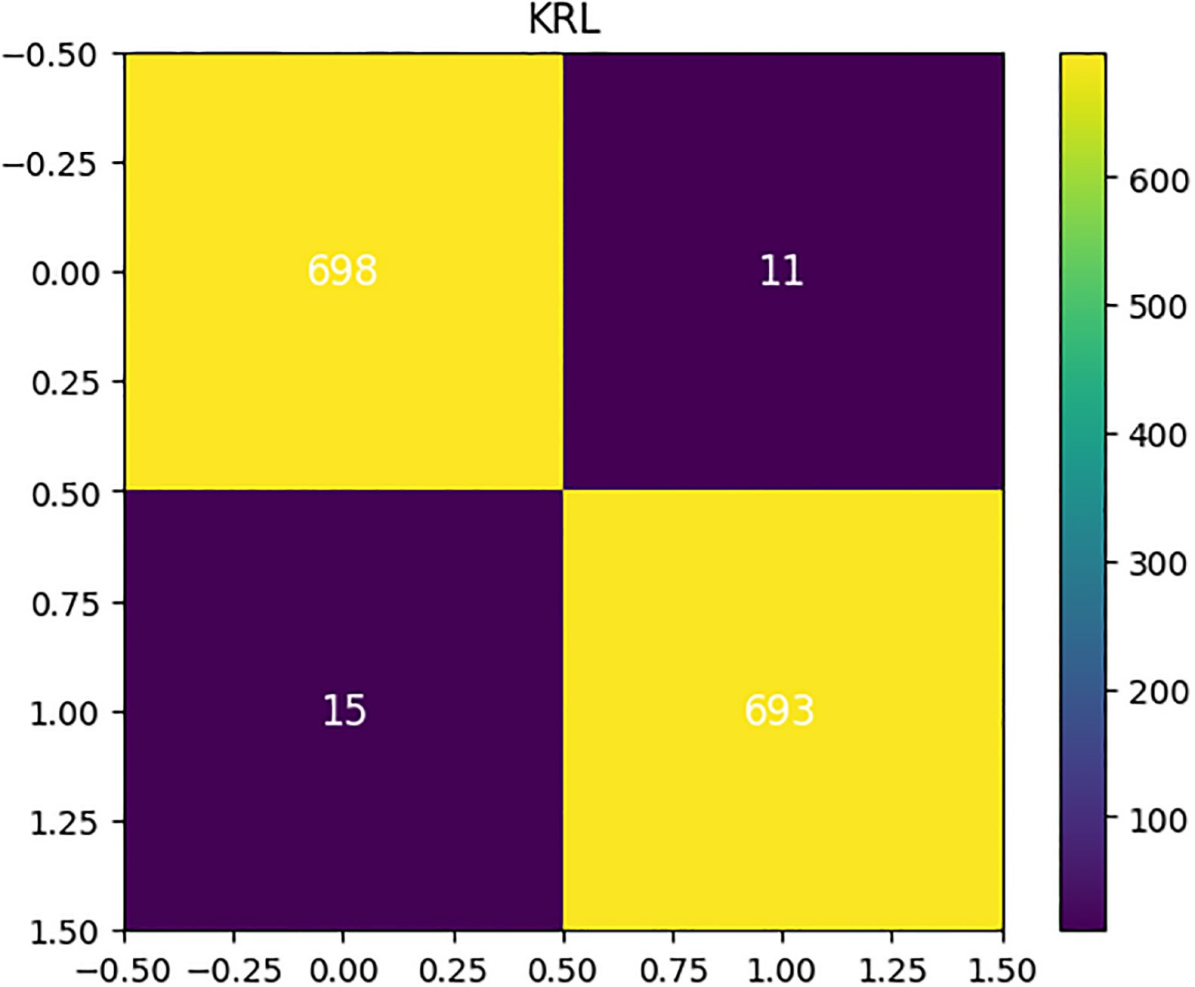
The confusion matrix results analysis of the novel proposed meta-learning model.

In addition, we have validated the effectiveness of the novel proposed model through confusion matrix analysis, as illustrated in Fig 7. The results analysis shows that the proposed model achieved 26 correct predictions, validating its effectiveness compared to other applied methods. Our innovative model demonstrated superior performance scores when compared to alternative approaches for diagnosing thyroid conditions.

**Fig 8:**
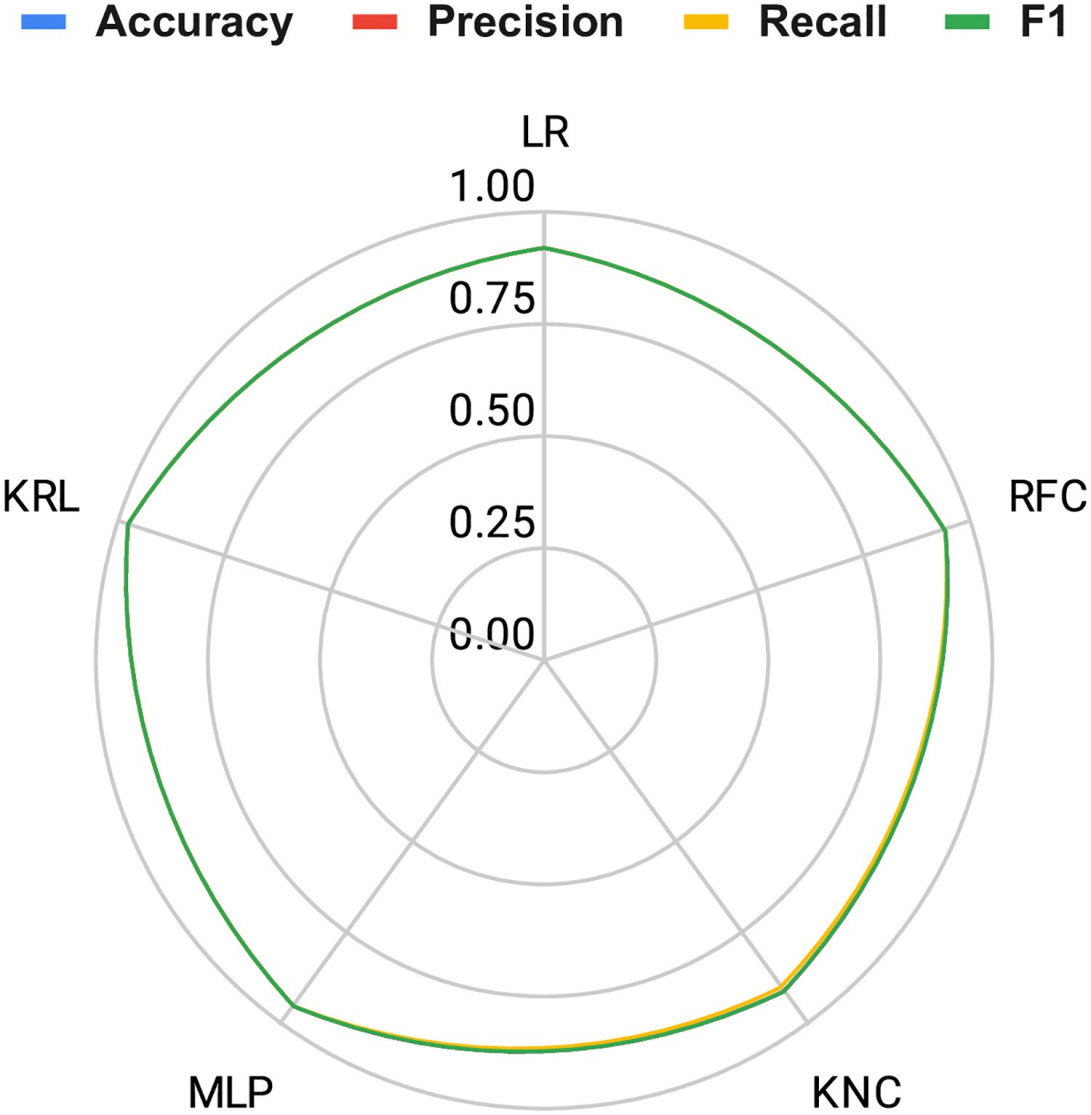
A performance evaluation using radar charts to compare the effectiveness of the proposed method against other applied methods.

The performance comparison among the applied methods and the proposed approach is visually depicted in Fig 8 using a radar chart. A comprehensive analysis of the chart reveals that the proposed KRL outperforms other methods by covering a significantly larger area on the radar plot. The graphical depiction emphasizes the superior efficacy of the proposed approach, substantiated by consistently elevated scores in performance evaluations.

### K-fold cross-validation analysis

In this section, we present the analysis of performance validation for the newly introduced meta-learning KRL approach in Table 6, utilizing k-fold cross-validation. Employing k-fold validation enhances the robustness of model performance estimates by mitigating the impact of data variations and, generously, facilitates a more comprehensive evaluation of the model’s capabilities. We opted for a 10-fold data split for result evaluations. The proposed meta-study model exhibited an impressive average k-fold accuracy of 0.98. The analysis conclusively demonstrates that the composite features, utilizing the KRL preparation, achieved a noteworthy score of 0.98, coupled with a lowest standard deviation of (+/-) 0.0038. This outcome underscores the reliability and consistency of the proposed novel meta-learning KRL approach in producing accurate and stable results.

**Table 6:** The 10-fold-based performance validations of the novel proposed KRL approach.

Folds	10-fold Accuracy
1	0.98
2	0.98
3	0.97
4	0.96
5	0.97
6	0.98
7	0.98
8	0.97
9	0.98
10	0.98
**Average**	**0.98**
**Standard Deviation**	**(+/-) 0.0038**

### State of the art comparisons

The state-of-the-art comparison for a fair analysis is performed in this section. We compare our proposed method with previous studies that utilized thyroid data, as depicted in Table 7. Earlier authors employed classical machine-learning approaches for thyroid disease classification. The analysis demonstrates that our proposed method excels in diagnosing thyroid diseases compared to existing models, achieving a remarkable accuracy of 0.98. This finding bridges the gap identified in prior research, addressing issues related to low-performance scores.

**Table 7:** The State of the art performance comparisons with recent studies.

Ref.	Proposed Approach	Performance Score
[11]	Random forest algorithm	94%
[12]	Support vector machine	93%
[14]	Random forest algorithm	94%
[15]	k-Nearest Neighbors	95%
[39]	SAPPHIRE	94%
[40]	PVP predictors	84%
**Our**	**Novel KRL**	**98%**

### Computational experience

The runtime computational analysis for thyroid detection reveals notable variations among the methods employed, as shown in Table 8. RF and KNC exhibit significantly lower computational scores of 0.05101 and 0.04026 seconds, respectively, suggesting their efficiency in processing thyroid data. In contrast, the KRL approach demonstrates a slightly higher computational score of 0.83311 seconds. However, KRL achieved high accuracy in performing thyroid detection.

**Table 8:** The runtime computational score analysis.

Method	Runtime computational Score (Seconds)
LR	0.46797
RF	0.05101
KNC	0.04026
KRL	0.83311

### Discussions

This study employed a novel meta-learning-based stacking method that yielded valuable results in diagnosing thyroid syndrome. By utilizing a combination of diverse study methods and meta-analyses, the proposed method was compared to medication, providing a more accurate assessment. The system integrates predictions from various models, demonstrating its effectiveness in identifying patterns in thyroid syndrome data, showcasing robust patient data, and illustrating its capability to enhance decision-making in the detection of thyroid disease. The preprocessed dataset is input into KNN and RF methods for prediction. The prediction outputs from these two separate groups are then combined and fed into a logistic-based new meta-learner to generate the final class prediction. The logistic regression-based meta-learner capitalizes on the strengths of each apporach in the set by utilizing their outputs as inputs. The advantage of adopting this new meta-study-based stacking method for diagnosing thyroid syndrome lies in its ability to enhance performance accuracy and efficiency in thyroid diagnosis.

## Conclusion and future direction

This study explores the detection of thyroid syndrome through the utilization of a meta-learning approach. Thyroid disorders cover a range of conditions that impact the thyroid gland, a crucial yet compact organ located in the neck. The dataset for thyroid diseases, containing 3773 samples collected from Kaggle, is employed to conduct experiments. A balanced dataset of 7,000 patient records, generated using SMOTE, is utilized for training various machine learning methods. A novel stacked model, the KRL approach, is proposed and evaluated using thyroid features. Four advanced machine-learning approaches are employed for thyroid detection. We conducted comparisons using four advanced machine-learning approaches. The performance of these methods is enhanced through hyperparameter tuning and validated using k-fold cross-validation. Extensive experiments present that the novel proposed KRL approach achieved a k-fold accuracy score of 0.98.

### Future work

In the future, we will build a GUI-based platform for real-time diagnosis of thyroid syndromes in hospitals. In addition, we will apply more advanced neural network-based approaches to enhance the performance scores for thyroid syndrome detection.

## Supporting information

S1 Appendix (PDF)

## References

[1] Lee HJ, Stefan-Lifshitz M, Li CW, Tomer Y. Genetics and epigenetics of autoimmune thyroid diseases: Translational implications. Best Practice & Research Clinical Endocrinology & Metabolism. 2023;37(2):101661. doi: 10.1016/j.beem.2022.101661 35459628 PMC9550878

[2] Martínez-Hernández R, Marazuela M. MicroRNAs in autoimmune thyroid diseases and their role as biomarkers. Best Practice & Research Clinical Endocrinology & Metabolism. 2023;37(2):101741. doi: 10.1016/j.beem.2023.101741 36801129

[3] Hollywood J, Hutchinson D, Feehery-Alpuerto N, Whitfield M, Davis K, Johnson L. The Effects of the paleo diet on autoimmune thyroid disease: a mixed methods review. Journal of the American Nutrition Association. 2023;42(8):727–736. doi: 10.1080/27697061.2022.2159570 36598468

[4] Aversano L, Bernardi ML, Cimitile M, Maiellaro A, Pecori R. A systematic review on artificial intelligence techniques for detecting thyroid diseases. PeerJ Computer Science. 2023;9:e1394. doi: 10.7717/peerj-cs.1394 37346658 PMC10280452

[5] Mohan E, Saravanan P, Natarajan B, Kumer SVA, Sambasivam G, Kanna GP, et al. Thyroid Detection and Classification Using DNN Based on Hybrid Meta-Heuristic and LSTM Technique. IEEE Access. 2023;11:68127–68138. doi: 10.1109/ACCESS.2023.3289511

[6] El-Hassani FZ, Fatih F, Joudar NE, Haddouch K. Deep Multilayer Neural Network with Weights Optimization-Based Genetic Algorithm for Predicting Hypothyroid Disease. Arabian Journal for Science and Engineering. 2023; p. 1–24.

[7] Zhang X, Wang X, Hu H, Qu H, Xu Y, Li Q. Prevalence and Trends of Thyroid Disease Among Adults, 1999-2018. Endocrine Practice. 2023;29(11):875–880. doi: 10.1016/j.eprac.2023.08.006 37619827

[8] Zhang J, Li J, Zhu Y, Fu Y, Chen L. Thyroidkeeper: a healthcare management system for patients with thyroid diseases. Health Information Science and Systems. 2023;11(1):49. doi: 10.1007/s13755-023-00251-w 37860050 PMC10582002

[9] Dondi F, Gatta R, Treglia G, Piccardo A, Albano D, Camoni L, et al. Application of radiomics and machine learning to thyroid diseases in nuclear medicine: a systematic review. Reviews in Endocrine and Metabolic Disorders. 2023; p. 1–12. doi: 10.1007/s11154-023-09822-4 37434097 PMC10808150

[10] Aversano L, Bernardi ML, Cimitile M, Iammarino M, Macchia PE, Nettore IC, et al. Thyroid disease treatment prediction with machine learning approaches. Procedia Computer Science. 2021;192:1031–1040. doi: 10.1016/j.procs.2021.08.106

[11] Alyas T, Hamid M, Alissa K, Faiz T, Tabassum N, Ahmad A. Empirical method for thyroid disease classification using a machine learning approach. BioMed Research International. 2022;2022. doi: 10.1155/2022/9809932 35711517 PMC9197629

[12] Prasad V, Rao TS, Babu MSP. Thyroid disease diagnosis via hybrid architecture composing rough data sets theory and machine learning algorithms. Soft Computing. 2016;20:1179–1189. doi: 10.1007/s00500-014-1581-5

[13] Poudel P, Illanes A, Ataide EJ, Esmaeili N, Balakrishnan S, Friebe M. Thyroid ultrasound texture classification using autoregressive features in conjunction with machine learning approaches. IEEE Access. 2019;7:79354–79365. doi: 10.1109/ACCESS.2019.2923547

[14] Butt AH, Alkhalifah T, Alturise F, Khan YD. Ensemble Learning for Hormone Binding Protein Prediction: A Promising Approach for Early Diagnosis of Thyroid Hormone Disorders in Serum. Diagnostics. 2023;13(11):1940. doi: 10.3390/diagnostics13111940 37296792 PMC10252793

[15] Savcı E, Nuriyeva F. DIAGNOSIS OF THYROID DISEASE USING MACHINE LEARNING TECHNIQUES. Journal of Modern Technology & Engineering. 2022;7(2).

[16] Rao AR, Renuka B. A machine learning approach to predict thyroid disease at early stages of diagnosis. In: 2020 IEEE international conference for innovation in technology (INOCON). IEEE; 2020. p. 1–4.

[17] Duggal P, Shukla S. Prediction of thyroid disorders using advanced machine learning techniques. In: 2020 10th International Conference on Cloud Computing, Data Science & Engineering (Confluence). IEEE; 2020. p. 670–675.

[18] Vadhiraj VV, Simpkin A, O’Connell J, Singh Ospina N, Maraka S, O’Keeffe DT. Ultrasound image classification of thyroid nodules using machine learning techniques. Medicina. 2021;57(6):527. doi: 10.3390/medicina57060527 34074037 PMC8225215

[19] Joshi AA, Aziz RM. Deep learning approach for brain tumor classification using metaheuristic optimization with gene expression data. International Journal of Imaging Systems and Technology. 2023; p. e23007.

[20] Yaqoob A, Verma NK, Aziz RM. Optimizing gene selection and cancer classification with hybrid sine cosine and cuckoo search algorithm. Journal of Medical Systems. 2024;48(1):10. doi: 10.1007/s10916-023-02031-1 38193948

[21] Rahman RU, Singh K, Tomar DS, Musheer R. Building Resilient Digital Forensic Frameworks for NoSQL Database: Harnessing the Blockchain and Quantum Technology. In: Sustainable Security Practices Using Blockchain, Quantum and Post-Quantum Technologies for Real Time Applications. Springer; 2024. p. 205–238.

[22] AYINDE B. Thyroid Sickness Determination;. https://www.kaggle.com/datasets/bidemiayinde/thyroid-sickness-determination.

[23] Elreedy D, Atiya AF, Kamalov F. A theoretical distribution analysis of synthetic minority oversampling technique (SMOTE) for imbalanced learning. Machine Learning. 2023; p. 1–21.37363047

[24] Raza A, Rustam F, Siddiqui HUR, Diez IdlT, Ashraf I. Predicting microbe organisms using data of living micro forms of life and hybrid microbes classifier. Plos one. 2023;18(4):e0284522. doi: 10.1371/journal.pone.0284522 37079536 PMC10118187

[25] Siddiqui HUR, Nawaz S, Saeed MN, Saleem AA, Raza MA, Raza A, et al. Footwear-integrated force sensing resistor sensors: A machine learning approach for categorizing lower limb disorders. Engineering Applications of Artificial Intelligence. 2024;127:107205. doi: 10.1016/j.engappai.2023.107205

[26] Raza A, Qadri AM, Akhtar I, Samee NA, Alabdulhafith M. LogRF: An Approach to Human Pose Estimation Using Skeleton Landmarks for Physiotherapy Fitness Exercise Correction. IEEE Access. 2023;11:107930–107939. doi: 10.1109/ACCESS.2023.3320144

[27] Raza A, Akhtar I, Abualigah L, Zitar RA, Sharaf M, Daoud MS, et al. Preventing Road Accidents Through Early Detection of Driver Behavior Using Smartphone Motion Sensor Data: An Ensemble Feature Engineering Approach. IEEE Access. 2023;11:138457–138471. doi: 10.1109/ACCESS.2023.3340304

[28] Thalji N, Raza A, Islam MS, Samee NA, Jamjoom MM. AE-Net: Novel Autoencoder-Based Deep Features for SQL Injection Attack Detection. IEEE Access. 2023;11:135507–135516. doi: 10.1109/ACCESS.2023.3337645

[29] Khalid M, Raza A, Younas F, Rustam F, Villar MG, Ashraf I, et al. Novel Sentiment Majority Voting Classifier and Transfer Learning-based Feature Engineering for Sentiment Analysis of Deepfake Tweets. IEEE Access. 2024;. doi: 10.1109/ACCESS.2024.3398582

[30] Sayed MS, Rony MAT, Islam MS, Raza A, Tabassum S, Daoud MS, et al. A Novel Deep Learning Approach for Forecasting Myocardial Infarction Occurrences with Time Series Patient Data. Journal of Medical Systems. 2024;48(1):53. doi: 10.1007/s10916-024-02076-w 38775899

[31] Younas F, Raza A, Thalji N, Abualigah L, Zitar RA, Jia H. An efficient artificial intelligence approach for early detection of cross-site scripting attacks. Decision Analytics Journal. 2024;11:100466. doi: 10.1016/j.dajour.2024.100466

[32] Raza A, Munir K, Almutairi MS, Sehar R. Novel Class Probability Features for Optimizing Network Attack Detection With Machine Learning. IEEE Access. 2023;11:98685–98694. doi: 10.1109/ACCESS.2023.3313596

[33] Darawsheh SR, Al-Shaar AS, Haziemeh FA, Alshurideh MT. Classification Thyroid Disease Using Multinomial Logistic Regressions (LR). In: The Effect of Information Technology on Business and Marketing Intelligence Systems. Springer; 2023. p. 645–659.

[34] Rajput G, Alashetty A. Diabetes Classification Using ML Algorithms. In: Inventive Systems and Control: Proceedings of ICISC 2023. Springer; 2023. p. 867–877.

[35] Basha MSA, Prabhavathi C, Khangembam V, Sucharitha MM, Oveis PM. Predicting Graduate Admissions using Ensemble Machine Learning Techniques: A Comparative Study of Classifiers and Regressors. In: 2023 2nd International Conference for Innovation in Technology (INOCON). IEEE; 2023. p. 1–6.

[36] Ashraf E, Kabeel A, Elmashad Y, Ward SA, Shaban WM. Predicting solar distiller productivity using an AI Approach: Modified genetic algorithm with Multi-Layer Perceptron. Solar Energy. 2023;263:111964. doi: 10.1016/j.solener.2023.111964

[37] Sathish T, Sunagar P, Singh V, Boopathi S, Al-Enizi AM, Pandit B, et al. Characteristics estimation of natural fibre reinforced plastic composites using deep multi-layer perceptron (MLP) technique. Chemosphere. 2023; p. 139346. doi: 10.1016/j.chemosphere.2023.139346 37379988

[38] Demir E, Bilgin M. Sentiment Analysis from Turkish News Texts with BERT-Based Language Models and Machine Learning Algorithms. In: 2023 8th International Conference on Computer Science and Engineering (UBMK). IEEE; 2023. p. 01–04.

[39] Charoenkwan P, Schaduangrat N, Moni MA, Manavalan B, Shoombuatong W, et al. SAPPHIRE: A stacking-based ensemble learning framework for accurate prediction of thermophilic proteins. Computers in Biology and Medicine. 2022;146:105704. doi: 10.1016/j.compbiomed.2022.105704 35690478

[40] Kabir M, Nantasenamat C, Kanthawong S, Charoenkwan P, Shoombuatong W. Large-scale comparative review and assessment of computational methods for phage virion proteins identification. EXCLI journal. 2022;21:11. doi: 10.17179/excli2021-4411 35145365 PMC8822302

