# Supplementary material for: A novel meta learning based stacked approach for diagnosis of thyroid syndrome
Source: PLoS One. 2024 Nov 1;19(11):e0312313. doi: 10.1371/journal.pone.0312313 (PMC11530063; doi:10.1371/journal.pone.0312313)
Supplement: S1 Appendix — (PDF) [file pone.0312313.s001.pdf]

Appendix

Table 9 contains the appendix.

Table 9. Nomenclature

Symbol	Definition
$\hat{y}$	model class prediction
$\in$	belong to
$\mathcal{N}_k$	number of choice k
β	representing prediction successes
(+/-)	standard deviations
